# Combining FMEA with DEMATEL models to solve production process problems

**DOI:** 10.1371/journal.pone.0183634

**Published:** 2017-08-24

**Authors:** Sang-Bing Tsai, Jie Zhou, Yang Gao, Jiangtao Wang, Guodong Li, Yuxiang Zheng, Peng Ren, Wei Xu

**Affiliations:** 1 Zhongshan Institute, University of Electronic Science and Technology of China, Guangdong, China; 2 Economics and Management College, Civil Aviation University of China, Tianjin, China; 3 Business and Law School, Foshan University, Guangdong, China; 4 School of Economics & Management, Shanghai Maritime University, Shanghai, China; 5 College of Tourism and Service Management, Nankai University, Tianjin, China; 6 School of Business, Dalian University of Technology, Panjin, China; 7 Business School, Nankai University, Tianjin, China; Southwest University, CHINA

## Abstract

Failure mode and effects analysis (FMEA) is an analysis tool for identifying and preventing flaws or defects in products during the design and process planning stage, preventing the repeated occurrence of problems, reducing the effects of these problems, enhancing product quality and reliability, saving costs, and improving competitiveness. However, FMEA can only analyze one influence factor according to its priority, rendering this method ineffective for systems containing multiple FMs whose effects are simultaneous or interact with one another. Accordingly, when FMEA fails to identify the influence factors and the factors being influenced, the most crucial problems may be placed in lower priority or remain unresolved. Decision-Making Trial and Evaluation Laboratory (DEMATEL) facilitates the determination of cause and effect factors; by identifying the causal factors that should be prioritized, prompt and effective solutions to core problems can be derived, thereby enhancing performance. Using the photovoltaic cell manufacturing industry in China as the research target, the present study combined FMEA with DEMATEL to amend the flaws of FMEA and enhance its effectiveness. First, FMEA was used to identify items requiring improvement. Then, DEMATEL was employed to examine the interactive effects and causal relationships of these items. Finally, the solutions to the problems were prioritized. The proposed method effectively combined the advantages of FMEA and DEMATEL to facilitate the identification of core problems and prioritization of solutions in the Chinese photovoltaic cell industry.

## Introduction

Failure mode and effects analysis (FMEA) is an analysis method for systematic operations and a component of total quality management. It is a dynamic analysis and early prevention tool aimed at identifying potential failure modes (FM) in a specific scope of systematic operations and classifying these potential FMs based on their influence levels to confirm their impact on the system[1]. FMEA is widely applied in the manufacturing industry to analyze the various stages of a product’s lifecycle or provide preventative analysis for new products or engineering design processes[2–3].

The United States has endeavored to standardize FMEA since the 1970s. Later, FMEA became widely used in the Japanese manufacturing sector[4]. The purpose of FMEA in a planned manufacturing process is to convert design characteristics into clearly defined operating conditions and guarantee that the outcomes and performance of the final product satisfy client demands and expectations[5–6]. Once a potential FM or failure effect is identified, corrective measures can be implemented to eliminate the potential FM or continue improving operations—thereby reducing the severity and frequency of the potential FM and improving detection—and to standardize the basic operations and regulations in the planned process, which can serve as a reference for future preventative and technical actions.

FMEA techniques are widely applied in design and process management. The preventative analysis method of FMEA for structured systems is advantageous in that (1) it is easy to understand and operate; (2) it is fundamentally a qualitative analysis method that can also be employed for quantitative purposes; (3) it can prioritize FMs based on the risk priority numbers (RPNs) assigned to the risk factors of product designs and manufacturing processes, and engage in improvement actions based on prioritization. However, FMEA resolves factor-related problems by considering only one individual factor at a time based on its ranking. Analysis is difficult when multiple FMs interact or exert effects simultaneously, such that FMEA fails to identify which are the influence factors and which factors are being influenced. As a result, the most crucial problems may not be prioritized[7–9]. Decision-Making Trial and Evaluation Laboratory (DEMATEL) is characterized by its use of matrix operations to calculate factors’ causal relationships and extent of influence, structuralizing complex problems through the use of a causal map to determine the basic nature of the problem and thereby identify the core problem and facilitate subsequent solutions. DEMATEL can be adopted to classify factors into causal and effect factors. In addition, by ranking or prioritizing the causal factors, core problems can be resolved promptly and efficiently to enhance performance. Accordingly, this study combined FMEA and DEMATEL to analyze and resolve production problems through the strengths of both methods.

In response to the increasing prevalence of global warming, countries should not only regulate greenhouse gas emissions but also develop alternative energy models by eliminating carbon emissions from energy systems. Common energy sources such as natural gas, petroleum, and other fossil fuels release carbon dioxide into the atmosphere during combustion and expedite global warming. To stop global warming and reduce the damage to the ozone layer, governments and enterprises are becoming increasingly dependent on renewable energy sources such as solar power, wind power, water power, and bioenergy. This increased reliance has actuated the exponential growth of the photovoltaic industry in recent years. Using the photovoltaic (PV) cell manufacturing industry in China as the research target, the present study combined FMEA with a DEMATEL model to identify the core problems in the manufacturing process of PV cells and prioritize solutions to these problems.

FMEA and DEMATEL were combined to address the flaws of FMEA and enhance its effectiveness[10–12]. First, FMEA was used to identify items requiring improvement. Then, DEMATEL was employed to examine the interactive effects and causal relationships of these items. Finally, solutions to problems in the production process were prioritized. The proposed method effectively combined the advantages of FMEA and DEMATEL to facilitate the identification of core problems and prioritize solutions.

## Literature review

### Development of the Chinese photovoltaic cell industry

China possesses rich resources for generating solar energy, with two-third of local regions each exhibiting an annual sunshine duration of >2,200 hours and a radiant emittance of 120–280 W/m^2^, which equates to an annual irradiance level of >5,000 million J/m^2^, or 170 kg of standard coal equivalent. In total, terrestrial irradiance in China yields an annual energy level of 2.4 trillion metric ton of standard coal equivalent, approximating the total generation of ten thousand of the Three Gorges Dam[13–14]. Therefore, China is considered a favorable country for generating solar energy. With its endowment of natural resources, China has constructed demonstration sites for solar energy generation and implemented measures such as prioritizing them in the budgets of local governments. To increase incentives for installing PV cells, the government provides subsidies to encourage solar energy production[15]. Overall, China possesses a complete industrial chain for PV applications and massive domestic demand. This facilitates developing related end systems and accelerating the prevalence of PV cells, thereby enhancing the country’s sustainable development.

The Chinese economic reform has led to substantial economic growth in China, enabling it to surpass Japan as the second largest economic worldwide. However, China’s energy utilization and greenhouse gas emission rates have also surpassed that of the United States and is currently ranked first globally. Developing clean energy sources is thus imperative to China. Not only is China now ranked first in renewable energy production, it has also surpassed Germany as the global leader of solar energy generation according to a 2015 statistical report[16–17].

China possesses the largest PV cell market worldwide. Since 2013, the country has become the global leader in PV cell installation. The Chinese PV cell industry continues to expand, now comprising more than 400 firms[18]. In 2015, China became the largest producer of PV energy. However, its generation per person was still lower than that of Germany, Japan, or the United States. According to the National Energy Administration, the PV installed capacity in China was increased by 34.54 GW in 2016, enabling the accumulated PV installed capacity to reach 77.42 GW; both the extent of increase and accumulated capacity ranked first worldwide^18-19^. Currently, solar energy only accounts for 1% of the annual total electricity output of China. The National Energy Administration plans to increase the PV installed capacity by 110 million kW by 2020. In 2030, the total consumption ratio of non-fossil fuels in China is expected to increase from 11% to 20%[19–20].

### Development and application of FMEA

FMEA was first applied by Grumman Aircraft Corporation to analyze the FMs in flight control systems. The effectiveness of FMEA gradually gained recognition, leading to its expansion from military aviation to general military applications FMEA focuses on early prevention, eliminating quality differences, and maintaining product stability, while reducing material waste, defective products, and discharge waste[10–11]. FMEA has become an indicator of ability and eligibility among the three largest automotive manufacturers in the world. Moreover, it was listed as a standard and essential analysis tool in QS9000. FMEA has expanded into industrial applications in recent years. It is now considered an international standard and an essential analysis method in the development of various industrial products.

FMEA is a preventative analysis tool used in product design and process planning to help users identify flaws and potential defects in products or process designs, thereby preventing the repeated occurrence of problems, reducing the effects of these problems, enhancing product quality and reliability, saving costs, and improving competitiveness.

FMEA can be categorized into system FMEA, design FMEA (DFMEA), process FMEA (PFMEA), and functional FMEA when applied in the system development, product design, production process, or after-sales stages, respectively^11-12^. DFMEA and PFMEA are the most commonly applied types and were incorporated in QS9000. DFMEA is applied in the design and conceptualization stage to review system and component structures and functional problems and formulate measures to prevent the occurrence of problems. PFMEA is applied before production commences or during quality planning to predict poor processes and review early prevention measures in the process design stage. It involves the systematic review and analysis of new or modified processes to predict, resolve, and track potential problems within a specific process.

### DEMATEL application

DEMATEL was introduced in the Science and Human Affairs Program of the Battelle Memorial Institute of Geneva in 1971. During the early stages of development, DEMATEL was applied primarily to resolve complex global problems such as race, hunger, environmental protection, and energy. The three main research domains were (1) examining global problem structures; (2) analyzing complex global problems and developing suitable solutions; and (3) reviewing studies, models, and data concerning global problems[21–22].

**Fig 1:**
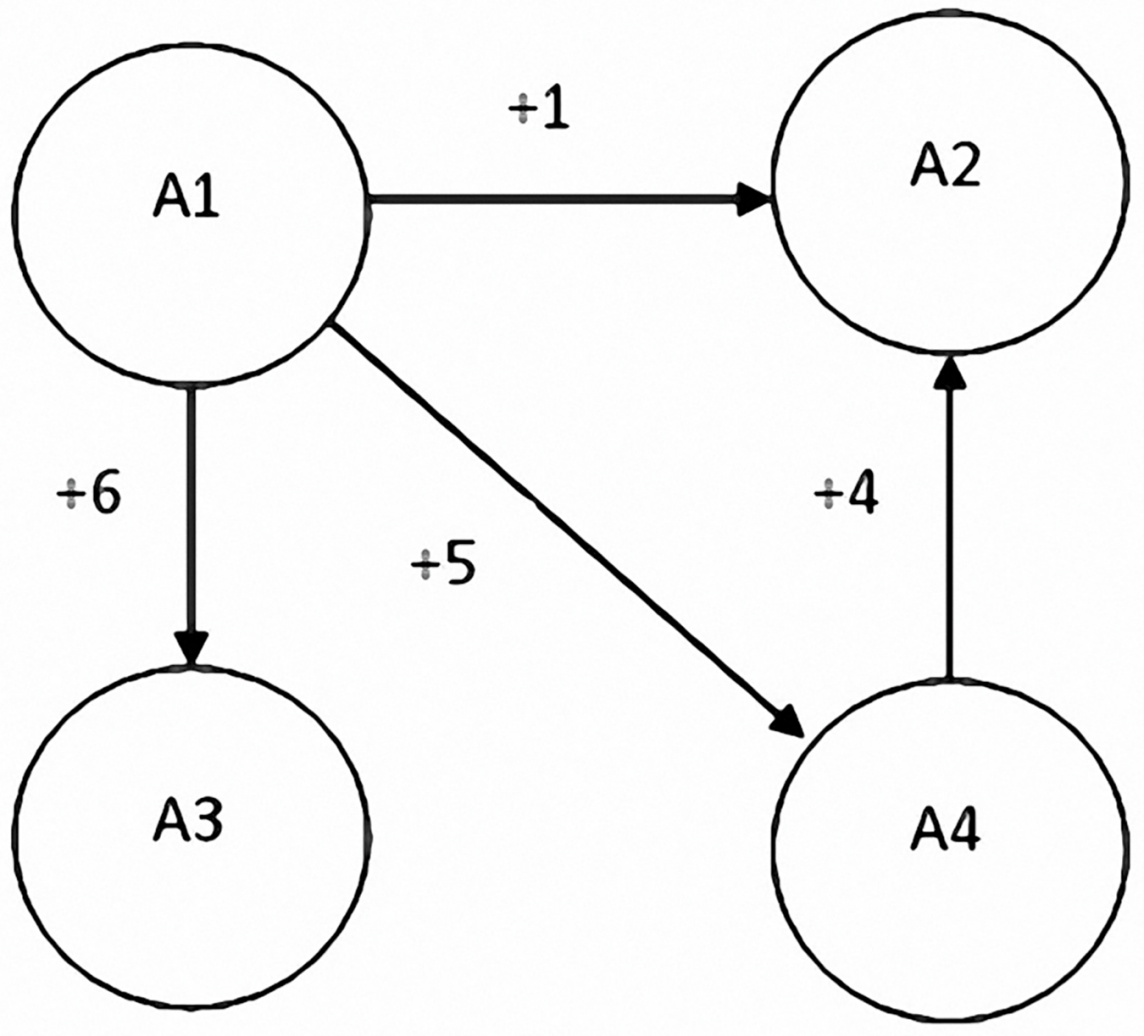
The Affect relationship diagram.

DEMATEL is characterized by its use of matrix operations to calculate factors’ causal relationships and extent of influence. Through a relationship map, DEMATEL explains the extent of influence and direction of influence caused by each factor, with the numbers indicating the extent of influence (Fig 1). By structuralizing the problem, criteria can be classified into cause and effect groups to clarify the nature of the problem, in turn identifying the core problem and corresponding solutions[23–24].

Lee et al. [25]asserted that the main feature of DEMATEL is its application of matrix operations to highlight the causal relationships and extent of influence between factors. A cause-and-effect diagram is then illustrated to structure complex problems and clarify the nature of the problems, facilitating the identification of core problems and the formulation of improvement strategies. Lee et al.[25] and Tsai et al. [26]maintained that when DEMATEL is applied, the analysis factors must satisfy several assumptions.

1. Clear problem properties: During the problem formation and planning stage, the properties of the research problem must be confirmed to ensure the solution is accurately established.
2. Clear problem relationship: The relationships between each factor and all other factors in the problem must be determined; the extent of correlation can be expressed using a rating system of 0 to 4.
3. Clear factor qualities: Each factor should be defined based on the relevant problem, and a consensus must be determined for these descriptions.

## Methods

### Risk priority number

RPN is generally used in FMEA methods to identify key FMs. The quantitative evaluation results show the relative importance of each potential FM, which can be used to prioritize improvement measures[12]. The RPN calculation method proposed by the Automotive Industry Action Group is used to prioritize failure risks. The professional knowledge and practical experience of industry experts or quality control teams are collected and applied to rate and score risk factors based on severity, occurrence, and detection. The three scores for each factor are multiplied to obtain the RPN score. The most urgent risk factor is the factor with the highest score[4].

RPNi=Si*Oi*Di

where Si represents the severity of the *i*th factor, Oi represents the occurrence of the *i*th factor, and Di represents the detection of the *i*th factor.

1. Severity Evaluation:
  The extent of the influence of FM on severity scores depends on the content of the project. The FMEA task force discusses each FM and allocates a score between 1 (lowest) and 10 (highest).
2. Occurrence Evaluation:
  Occurrence refers to the possibility of the FM occurring. The FMEA task force discusses each FM and allocates a score between 1 (lowest) and 10 (highest).
3. Detection Evaluation:
  The FMEA task force analyzes each potential FM to determine the possibility of its occurrence and evaluate whether the existing operating regulations can effectively identify and control each FM. During analysis, the task force assumes the potential FM has already occurred to determine whether the existing operating regulations can identify and control the FM. The FMEA task force then discusses each FM and allocates a score between 1 (lowest) and 10 (highest).

Once FM identification, failure effect analysis, and failure risk evaluation are completed, the FMEA task force can set a threshold value for the failure RPN. This value determines whether preventative and improvement measures should be prioritized to resolve failure risks and the order in which this should be conducted.

FMEA for structured systems is easy to understand and operate. Therefore, it is widely used in technology development and management, as well as in process management technologies. However, evaluations and outcomes are often tainted by subjectivity[27]. In addition, FMEA can only analyze one influence factor at a time, rendering this method ineffective for systems that contain multiple FMs with simultaneous effects or that interact with one another.

### DEMATEL model calculation process

DEMATEL clarifies the complex relationships between factors and provides solutions by comparing these factors in the system, using matrix operations to calculate the direct and indirect causal relationships and extent of influence, and quantifying the extent of mutual influence between factors.

The calculation procedures of DEMATEL can be summarized into the following steps[28–31]:

1. Establishing the measurement scale and determining the causal relationships
  List and define the various factors involved in a complex system through a literature review, brainstorming session, or expert survey. Design a scale to demonstrate the extent of influence of these factors and employ pair-wise comparison to elucidate the causal relationships between the factors.
2. Establishing a direct-relation matrix
  Once the scale is complete, invite experts to participate in a survey. Instruct the experts to engage in a pair-wise comparison to determine the presence and extent of influence relationships between the factors. Use the results to create a direct-relation matrix, where values in the matrix represent the extent of influence between the factors. Set the values on the diagonal line in the matrix to zero[32–34].
  $$X=\;\left[\begin{array}{cc} \begin{array}{cc} 0 & x_{12}\\ x_{21} & 0 \end{array} & \begin{array}{cc} \cdots & x_{1n}\\ \cdots & x_{2n} \end{array}\\ \begin{array}{cc} \vdots & \vdots \\ x_{n1} & x_{n2} \end{array} & \begin{array}{cc} \ddots & \vdots \\ \cdots & 0 \end{array} \end{array}\right]$$ (1)
3. Calculating the normalized direct-relation matrix
  Use the column vectors and maximum values as the baseline for normalization[35–36].
  $$\lambda =\frac{1}{{\text{max}}_{1\leq i\leq n}\sum_{j=1}^{n}x_{ij}}$$ (2)
  N=λX (3)
4. Calculating the direct/indirect-relation matrix (*T*) or the total-relation matrix
  $$T={\text{lim}}_{k\to \infty }\left(N+N^{2}+\cdots +N^{k}\right)=N{\left(I-N\right)}^{-1}$$ (4)
  where *I* represents the identity matrix.
5. Calculating the values in each row and column
  Sum the values in each row and column in the total-relation matrix (*T*). Let *D*_*i*_ be the sum of the *i*th column and *R*_*j*_*j* be the sum of the *j*th row. Thus, the *D*_*i*_ and *R*_*i*_ values comprise both indirect and direct influences.
  $$D_{i}=\sum_{j=1}^{n}t_{ij}\;\left(i=1,\;2,\;\cdots ,n\right)$$ (5)
  $$R_{i}=\sum_{i=1}^{n}t_{ij}\;\left(j=1,\;2,\;\cdots ,\;n\right)$$ (6)
6. Illustrating the DEMATEL cause-and-effect diagram
  Let (*D + R*) be prominence, which represents the total relationships between the cause and effect of specific criteria. This value represents the prominence of the criteria in the problem. Let (*D − R*) be relation, which represents the differences between the cause and effect of specific criteria. This value represents the causal relationships of the criteria in the problem, where a positive value denotes that the criteria contain greater cause characteristics and a negative value denotes that the criteria contain greater effect characteristics. The cause-and-effect diagram is llustrated using (*D + R*) as the horizontal axis and *(D − R*) as the vertical axis[37]. The diagram simplifies complex causal relationships into an easy-to-understand visual structure. Decision-makers can determine factor types based on the characteristics of the factors and formulate appropriate solutions based on the extent of influence of each factor.

Attribute *k* is either a cause or effect attribute when (*D*_*k*_ *− R*_*k*_) is a positive or negative value, respectively. The size of the (*D*_*k*_ *+ R*_*k*_) represents the extent of the attribute’s cause or effect. Based on the coordinates in (*D*_*k*_ *+ R*_*k*_) and (*D*_*k*_ *− R*_*k*_), *k* can be categorized into four categories[38–39]:

1. Positive (*D*_*k*_ *− R*_*k*_) and large (*D*_*k*_ *+ R*_*k*_) values: *k* is a cause factor and an actuating factor for solving the problem.
2. Positive (*D*_*k*_ *− R*_*k*_) and small (*D*_*k*_ *+ R*_*k*_) values: *k* is an independent factor and influences only a small number of other factors.
3. Negative (*D*_*k*_ *− R*_*k*_) and small (*D*_*k*_ *+ R*_*k*_) values: *k* is an independent factor and is influenced by only a small number of factors.
4. Negative (*D*_*k*_ *− R*_*k*_) and large (*D*_*k*_ *+ R*_*k*_) values: *k* is a core problem that requires resolution. However, it is an effect attribute, and thus it cannot be directly improved.

## Results and discussion

### FMEA results

Using the PV cell manufacturing industry in China as the research target, the present study combined FMEA with a DEMATEL model to identify the core problems in the manufacturing process of PV cells and prioritize the solutions to these problems.

**Fig 2:**
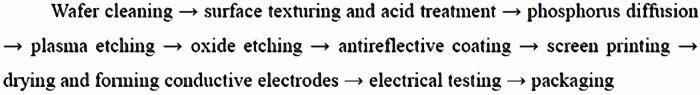
PV cell manufacturing process.

The process for manufacturing PV cells comprises 10 major components. In sequential order, the components are wafer cleaning, surface texturing and acid treatment, phosphorus diffusion, plasma etching, oxide etching, antireflective coating, screen printing, drying and forming conductive electrodes, electrical testing, and packaging (Fig 2). Among the components, screen printing is a most crucial step in the manufacturing process, and is also the component with the lowest yield. Therefore, screen printing was the focus of the present study.

Interviews were conducted with 20 experts in PV cell manufacturing, 15 of whom had more than 15 years of experience in the industry. Among these 15 experts, 3 were general managers, 6 were deputy general managers in the R&D or manufacturing sector, and 6 were factory managers. The remaining 5 experts were scholars specializing in the field of PV cells. This expert list was finalized following discussion of an initial list. The experts were visited and completed the questionnaires in person.

The interview results revealed 12 causes of failure in the screen printing stage of PV cell manufacturing: (a) screen deformation, (b) frame deformation, (c) suction positioning system failure, (d) uneven slurry viscosity, (e) lack of slurry, (f) slurry preparation error, (g) clean room temperature setting error, (h) clean room humidity setting error, (i) lack of cleanliness in clean rooms, (j) operation error, (k) parameter setting error, and (l) lack of staff proficiency. The 20 experts rated the causes of failure in terms of severity, occurrence, and detection by assigning scores of 1–10 for each item. The scores were then averaged and rounded to the nearest whole number (Table 1).

**Table 1:** FMEA analysis results.

Code	Cause of Failure	Severity Evaluation	Occurrence Evaluation	Detection Evaluation	RPN S*O*D	Order of Improvement
a	Screen deformation	9	8	4	288	**1**
b	Frame deformation	7	5	4	140	**2**
c	Suction positioning system failure	6	3	3	54	9
d	Uneven slurry viscosity	5	4	5	100	**5**
e	Lack of slurry	4	2	2	16	12
f	Slurry preparation error	6	4	4	96	**6**
g	Clean room temperature setting error	5	2	4	40	10
h	Clean room humidity setting error	4	2	4	32	11
i	Lack of cleanliness in clean room	4	6	3	72	8
j	Operation error	8	4	4	128	**4**
k	Parameter setting error	7	5	4	140	**2**
l	Lack of staff proficiency	7	4	3	84	7

The RPN of each failure item was calculated by averaging the scores provided by the experts. The items with the highest RPNs were (a), (b), (k), (j), (d), and (f). The results of a conventional FMEA showed that these six items were the key factors influencing process yield. Therefore, these items, particularly the first three, must be resolved to improve process yield.

### DEMATEL procedure

#### Questionnaire

The 12 causes of process failure served as indices in the development of the DEMATEL questionnaire. A 7-point scale was adopted for the scoring system, where 6 represented the highest effect and 0 represented no effect. The respondents to the DEMATEL questionnaire were the 20 experts that participated in the FMEA survey. The content was explained to the respondents before the questionnaires were administered. All the questionnaires were retrieved, for a retrieval rate of 100%.

#### Results

The expert survey results are tabulated in Table 2. The scores of the experts were averaged and rounded to the first decimal place to create a matrix of the 12 indices comprising 144 grids. Among the 144 grids, the 12 diagonal grids with zero influence were excluded, for a total of 132 grids that represented the mutual influence of the 12 factors.

**Table 2:** Initial direct-relation matrix *X*.

Index	a	b	c	d	e	f	g	h	i	j	k	l
a	0	4.3	5.4	4.1	0	0	0	0	0	0	0	0
b	2.8	0	2.9	0	0	0	0	0	0	0	0	0
c	2.2	2.4	0	0	0	0	0	0	0	0	0	0
d	0	0	0	0	3.2	0	0	0	0	0	0	0
e	0	0	0	1.8	0	0	0	0	0	0	0	0
f	0	0	0	5.7	4.5	0	0	0	0	2.5	2.4	0
g	0	0	0	0	0	0	0	1.7	2.2	0	0	0
h	0	0	0	0	0	0	2.3	0	0	0	0	0
i	0	0	0	0	0	0	3.3	3.9	0	0	0	0
j	0	0	0	0	0	0	2.1	3.1	0	0	2.9	0.8
k	0	0	0	0	0	0	3.2	3.2	0	0	0	0.3
l	0	0	0	0	0	2.3	3.2	2.4	1.8	4.4	5.1	0

Then, the direct-relation matrix was normalized using the column vectors and maximum values as the baseline, where *λ* was the maximum value for the sum of each column. Using Eq (2), the values in direct-relation matrix *X* were multiplied by *λ* to formulate the normalized direct-relation matrix *N* (Table 3).

**Table 3:** Normalized direct-relation matrix *N*.

Index	a	b	c	d	e	f	g	h	i	j	k	l
a	0.00	0.22	0.28	0.21	0.00	0.00	0.00	0.00	0.00	0.00	0.00	0.00
b	0.15	0.00	0.15	0.00	0.00	0.00	0.00	0.00	0.00	0.00	0.00	0.00
c	0.11	0.13	0.00	0.00	0.00	0.00	0.00	0.00	0.00	0.00	0.00	0.00
d	0.00	0.00	0.00	0.00	0.17	0.00	0.00	0.00	0.00	0.00	0.00	0.00
e	0.00	0.00	0.00	0.09	0.00	0.00	0.00	0.00	0.00	0.00	0.00	0.00
f	0.00	0.00	0.00	0.30	0.23	0.00	0.00	0.00	0.00	0.13	0.13	0.00
g	0.00	0.00	0.00	0.00	0.00	0.00	0.00	0.09	0.11	0.00	0.00	0.00
h	0.00	0.00	0.00	0.00	0.00	0.00	0.12	0.00	0.00	0.00	0.00	0.00
i	0.00	0.00	0.00	0.00	0.00	0.00	0.17	0.20	0.00	0.00	0.00	0.00
j	0.00	0.00	0.00	0.00	0.00	0.00	0.11	0.16	0.00	0.00	0.15	0.04
k	0.00	0.00	0.00	0.00	0.00	0.00	0.17	0.17	0.00	0.00	0.00	0.02
l	0.00	0.00	0.00	0.00	0.00	0.12	0.17	0.13	0.09	0.23	0.27	0.00

Eqs (3) and (4) were used to calculate the total-relation matrix *Tc* (Table 4).

**Table 4:** Total-relation matrix *T*.

Index	a	b	c	d	e	f	g	h	i	j	k	l
a	0.08	0.29	0.35	0.23	0.04	0.00	0.00	0.00	0.00	0.00	0.00	0.00
b	0.18	0.07	0.21	0.04	0.01	0.00	0.00	0.00	0.00	0.00	0.00	0.00
c	0.15	0.17	0.07	0.03	0.01	0.00	0.00	0.00	0.00	0.00	0.00	0.00
d	0.00	0.00	0.00	0.02	0.17	0.00	0.00	0.00	0.00	0.00	0.00	0.00
e	0.00	0.00	0.00	0.10	0.02	0.00	0.00	0.00	0.00	0.00	0.00	0.00
f	0.00	0.00	0.00	0.32	0.29	0.00	0.05	0.05	0.01	0.13	0.15	0.01
g	0.00	0.00	0.00	0.00	0.00	0.00	0.03	0.12	0.12	0.00	0.00	0.00
h	0.00	0.00	0.00	0.00	0.00	0.00	0.12	0.01	0.01	0.00	0.00	0.00
i	0.00	0.00	0.00	0.00	0.00	0.00	0.20	0.23	0.02	0.00	0.00	0.00
j	0.00	0.00	0.00	0.00	0.00	0.01	0.18	0.22	0.02	0.01	0.17	0.04
k	0.00	0.00	0.00	0.00	0.00	0.00	0.20	0.19	0.02	0.00	0.01	0.02
l	0.00	0.00	0.00	0.04	0.04	0.12	0.31	0.27	0.13	0.25	0.32	0.02

Eqs (5) and (6) were used to calculate the *D*_*i*_ values in each column and the *R*_*j*_ values in each row and to determine the prominence (D + R) and relation (D − R) of the indices (Table 5).

**Table 5:** Summary of the prominence and relation of the 12 indices.

Index	D	R	D + R	D—R
a	0.99	0.41	1.40	0.58
b	0.50	0.52	1.02	-0.01
c	0.42	0.63	1.04	-0.21
d	0.19	0.78	0.97	-0.60
e	0.11	0.56	0.67	-0.45
f	1.01	0.13	1.14	0.88
g	0.27	1.09	1.36	-0.82
h	0.15	1.09	1.24	-0.94
i	0.45	0.34	0.79	0.11
j	0.65	0.40	1.04	0.25
k	0.44	0.64	1.08	-0.20
l	1.49	0.08	1.58	1.41
Average			1.11	0.00

**Fig 3:**
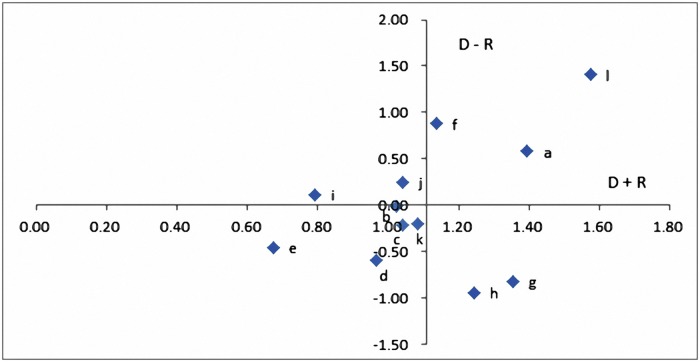
Relational diagram of the 12 indices.

Finally, a relation diagram of the 12 indices was illustrated using prominence as the horizontal axis and relation as the vertical axis (Fig 3).

Based on the results of Table 5 and Fig 2, the causal relationships of the 12 indices are listed below.

1. High prominence and high relation: The indices in this quadrant comprise (a), (f), and (l). These indices are the core cause factors influencing the other items. Thus, they are the actuating factors for solving problems.
2. Low prominence and high relation: The indices in this quadrant comprise (i) and (j) and slightly influence a few of the other indices. Thus, they are relatively independent.
3. Low prominence and low relation: The indices in this quadrant comprise (b), (c), (d), (e), and (k) and are slightly influenced by the other indices. Thus, they are relatively independent.
4. High prominence and low relation: The indices in this quadrant comprise (g) and (h) and are effect factors that are influenced by the other items. Although these indices require improvement, they are effect factors, and thus they cannot be directly improved.

In summary, (a), (f), and (l) are the three factors with high relation and high prominence, indicating that they influence the other indices. Improving these indices can effectively resolve core problems and incidentally resolve the unfavorable effects of the other indices.

### Combined discussion of FMEA and DEMATEL

The orders of improvement produced by FMEA and DEMATEL were independently discussed in previous sections. In this section, the two analysis methods were combined to facilitate the identification of core problems and determine the optimal order in which to improve them.

Through the results of the conventional FMEA, six factors were identified to significantly influence yield based on their RPNs. In sequential descending order, they were (a), (b), (k), (j), (d), and (f).

DEMATEL enables the identification of causal factors and ranks them to resolve core problems rapidly and efficiently and thereby enhance performance. By combing DEMATEL with FMEA analysis, we found that (a), (f), and (l) were the actual causal indicators; namely, they were the core items that influenced other indicators and were the driving factors of solutions. In other words, if the other factors such as (b), (k), (j), and (d) are addressed first rather than these three factors, production problems will continue to occur regardless of the solutions applied.

An in-depth analysis was conducted to determine the underlying reasons for the discrepancies between the two methods. The results indicated that (a) was likely to lead to (b) and (c) and that (f) was likely to cause (d) and (e), leading to poor-quality screen printing. In addition, (l) is the direct cause of (j) and (k).

The true reasons for process failure and the ideal order in which to solve various failure problems can be clearly identified by combining FMEA and DEMATEL, thereby effectively resolving process problems and enhancing production yield.

## Conclusion

FMEA is a preventative analysis tool used in product design and process planning to help users identify flaws and potential defects, thereby preventing the repeated occurrence of problems, reducing the effects of these problems, enhancing product quality and reliability, saving costs, and improving competitiveness.

FMEA resolves problems by addressing individual factors and prioritizing the factors that can be used for deriving solutions. When multiple FMs are at work or when they interact with one another, analysis becomes difficult, such that FMEA will incorrectly identify the influence factors and factors being influenced. Consequently, crucial problems may remain unresolved.

We combined FMEA and DEMATEL to address the flaws of FMEA and enhance its effectiveness. Therefore, FMEA was first used to identify the items requiring improvement, followed by applying DEMATEL to examining the causal relationships and extent of influence of the items identified. Finally, priority for resolving the core problems was suggested.

Selecting the PV cell manufacturing industry in China as the research target, the present study combined FMEA with DEMATEL to identify the core problems in the PV cell manufacturing process to prioritize the solutions to these problems.

In addition to contributing to academia, the method proposed in the present study can be implemented in industrial practice. Future researchers can examine a wider range of industries or adopt other evaluation methods for analysis and comparison.

## Supporting information

S1 File Questionnaire—Docs. (DOCX)
