# Supplementary material for: Combining FMEA with DEMATEL models to solve production process problems
Source: PLoS One. 2017 Aug 24;12(8):e0183634. doi: 10.1371/journal.pone.0183634 (PMC5570359; doi:10.1371/journal.pone.0183634)
Supplement: S1 File — (DOCX) [file pone.0183634.s001.docx]

**Production problems and solutions in the solar energy industry**

Research purpose:

Dear Sir or Madam,

Owing to your expertise and reputation in our field, we invite you to complete the following questionnaire for use in our study on production problems and solutions in the solar energy industry. We will use the findings from these expert questionnaire responses to clarify related information. We hope to rely on your experience to assist us in our research. Your responses will be used for academic purposes only. Thank you.

Prof. Sang-Bing Tsai

This questionnaire has two parts. Part 1: FMEA and RPN analysis of production factors in the solar energy industry. Part 2: Influence analysis of relationships between all production factors.

Part 1: FMEA and RPN analysis of production factors

Instructions:

1. Rate the following causes of failure in terms of severity, occurrence, and detection using scores ranging from 1 (lowest) to 10 (highest).

| Code | Cause of Failure | Severity Evaluation | Occurrence Evaluation | Detection Evaluation |
| --- | --- | --- | --- | --- |
| a | Screen deformation |  |  |  |
| b | Frame deformation |  |  |  |
| c | Suction positioning system failure |  |  |  |
| d | Uneven slurry viscosity |  |  |  |
| e | Lack of slurry |  |  |  |
| f | Slurry preparation error |  |  |  |
| g | Clean room temperature setting error |  |  |  |
| h | Clean room humidity setting error |  |  |  |
| i | Lack of cleanliness in clean room |  |  |  |
| j | Operation error |  |  |  |
| k | Parameter setting error |  |  |  |
| l | Lack of staff proficiency |  |  |  |

Part 2: Influence analysis of relationships between production factors—DEMATEL

Instructions:

1. According to your personal opinion, rate the influence relationships between the production factors using a 7-point Likert scale (6 being *highest influence*, 0 being *no influence*; items with medium scores will be evaluated according to priority).

1. Influence of screen deformation on other criteria

| Criteria | Screen deformation | Frame deformation | Suction positioning system failure | Uneven slurry viscosity | Lack of slurry | Slurry preparation error | Clean room temperature setting error | Clean room humidity setting error | Lack of cleanliness in clean room | Operation error | Parameter setting error | Lack of staff proficiency |
| --- | --- | --- | --- | --- | --- | --- | --- | --- | --- | --- | --- | --- |
| Screen deformation | No |  |  |  |  |  |  |  |  |  |  |  |

2. Influence of frame deformation on other criteria

| Criteria | Screen deformation | Frame deformation | Suction positioning system failure | Uneven slurry viscosity | Lack of slurry | Slurry preparation error | Clean room temperature setting error | Clean room humidity setting error | Lack of cleanliness in clean room | Operation error | Parameter setting error | Lack of staff proficiency |
| --- | --- | --- | --- | --- | --- | --- | --- | --- | --- | --- | --- | --- |
| Frame deformation |  | No |  |  |  |  |  |  |  |  |  |  |

3. Influence of suction positioning system failure on other criteria

| Criteria | Screen deformation | Frame deformation | Suction positioning system failure | Uneven slurry viscosity | Lack of slurry | Slurry preparation error | Clean room temperature setting error | Clean room humidity setting error | Lack of cleanliness in clean room | Operation error | Parameter setting error | Lack of staff proficiency |
| --- | --- | --- | --- | --- | --- | --- | --- | --- | --- | --- | --- | --- |
| Suction positioning system failure |  |  | No |  |  |  |  |  |  |  |  |  |

4. Influence of uneven slurry viscosity on other criteria

| Criteria | Screen deformation | Frame deformation | Suction positioning system failure | Uneven slurry viscosity | Lack of slurry | Slurry preparation error | Clean room temperature setting error | Clean room humidity setting error | Lack of cleanliness in clean room | Operation error | Parameter setting error | Lack of staff proficiency |
| --- | --- | --- | --- | --- | --- | --- | --- | --- | --- | --- | --- | --- |
| Uneven slurry viscosity |  |  |  | No |  |  |  |  |  |  |  |  |

5. Influence of lack of slurry on other criteria

| Criteria | Screen deformation | Frame deformation | Suction positioning system failure | Uneven slurry viscosity | Lack of slurry | Slurry preparation error | Clean room temperature setting error | Clean room humidity setting error | Lack of cleanliness in clean room | Operation error | Parameter setting error | Lack of staff proficiency |
| --- | --- | --- | --- | --- | --- | --- | --- | --- | --- | --- | --- | --- |
| Lack of slurry |  |  |  |  | No |  |  |  |  |  |  |  |

6. Influence of slurry preparation error on other criteria

| Criteria | Screen deformation | Frame deformation | Suction positioning system failure | Uneven slurry viscosity | Lack of slurry | Slurry preparation error | Clean room temperature setting error | Clean room humidity setting error | Lack of cleanliness in clean room | Operation error | Parameter setting error | Lack of staff proficiency |
| --- | --- | --- | --- | --- | --- | --- | --- | --- | --- | --- | --- | --- |
| Slurry preparation error |  |  |  |  |  | No |  |  |  |  |  |  |

7. Influence of clean room temperature setting error on other criteria

| Criteria | Screen deformation | Frame deformation | Suction positioning system failure | Uneven slurry viscosity | Lack of slurry | Slurry preparation error | Clean room temperature setting error | Clean room humidity setting error | Lack of cleanliness in clean room | Operation error | Parameter setting error | Lack of staff proficiency |
| --- | --- | --- | --- | --- | --- | --- | --- | --- | --- | --- | --- | --- |
| Clean room temperature setting error |  |  |  |  |  |  | No |  |  |  |  |  |

8. Influence of clean room humidity setting error on other criteria

| Criteria | Screen deformation | Frame deformation | Suction positioning system failure | Uneven slurry viscosity | Lack of slurry | Slurry preparation error | Clean room temperature setting error | Clean room humidity setting error | Lack of cleanliness in clean room | Operation error | Parameter setting error | Lack of staff proficiency |
| --- | --- | --- | --- | --- | --- | --- | --- | --- | --- | --- | --- | --- |
| Clean room humidity setting error |  |  |  |  |  |  |  | No |  |  |  |  |

9. Influence of lack of cleanliness in clean rooms on other criteria

| Criteria | Screen deformation | Frame deformation | Suction positioning system failure | Uneven slurry viscosity | Lack of slurry | Slurry preparation error | Clean room temperature setting error | Clean room humidity setting error | Lack of cleanliness in clean room | Operation error | Parameter setting error | Lack of staff proficiency |
| --- | --- | --- | --- | --- | --- | --- | --- | --- | --- | --- | --- | --- |
| Lack of cleanliness in clean room |  |  |  |  |  |  |  |  | No |  |  |  |

10. Influence of operational error on other criteria

| Criteria | Screen deformation | Frame deformation | Suction positioning system failure | Uneven slurry viscosity | Lack of slurry | Slurry preparation error | Clean room temperature setting error | Clean room humidity setting error | Lack of cleanliness in clean room | Operation error | Parameter setting error | Lack of staff proficiency |
| --- | --- | --- | --- | --- | --- | --- | --- | --- | --- | --- | --- | --- |
| Operation error |  |  |  |  |  |  |  |  |  | No |  |  |

11. Influence of parameter settings error on other criteria

| Criteria | Screen deformation | Frame deformation | Suction positioning system failure | Uneven slurry viscosity | Lack of slurry | Slurry preparation error | Clean room temperature setting error | Clean room humidity setting error | Lack of cleanliness in clean room | Operation error | Parameter setting error | Lack of staff proficiency |
| --- | --- | --- | --- | --- | --- | --- | --- | --- | --- | --- | --- | --- |
| Parameter setting error |  |  |  |  |  |  |  |  |  |  | No |  |

12. Influence of lack of staff proficiency on other criteria

| Criteria | Screen deformation | Frame deformation | Suction positioning system failure | Uneven slurry viscosity | Lack of slurry | Slurry preparation error | Clean room temperature setting error | Clean room humidity setting error | Lack of cleanliness in clean room | Operation error | Parameter setting error | Lack of staff proficiency |
| --- | --- | --- | --- | --- | --- | --- | --- | --- | --- | --- | --- | --- |
| Lack of staff proficiency |  |  |  |  |  |  |  |  |  |  |  | N0 |
